# Vascular age as a key for a team-based approach to manage blood pressure bridging community pharmacists and primary healthcare physicians—The TOGETHER trial

**DOI:** 10.3389/fpubh.2025.1723100

**Published:** 2026-01-14

**Authors:** Enrique Rodilla, Monika Aichberger, Florian Ardelt, Vicente J. Baixauli, Sergio Cinza-Sanjurjo, Kathrin Danninger, María S. Fernández-Alfonso, Luis García-Ortiz, Viktoria Gastens, Ana-Karen Guzmán-Aguayo, Bernhard Hametner, Antonia M. Jiménez, Valeria Kopytina, Cristina Lugones-Sánchez, Christopher C. Mayer, Zuri Montalar, Telmo Pereira, Valérie Santschi, Wolfgang Schimetta, Thomas Weber, TOGETHER-Project Research Group, Monika Aichberger, Florian Ardelt, Vicente Baixauli, Sergio Cinza, Kathrin Danninger, María Soledad Fernández, Viktoria Gastens, Luis García-Ortiz, Ana Karen Guzmán-Aguayo, Bernhard Hametner, Antonela Jiménez, Valeria Kopytina, Cristina Lugones-Sánchez, Christopher C. Mayer, Zuri Montalar, Telmo Pereira, Enrique Rodilla, Valérie Santschi, Wolfgang Schimetta, Thomas Weber

**Affiliations:** 1 Department of Internal Medicine, Hypertension and Vascular Risk Unit, Hospital de Sagunto, Valencia, Spain; 2 Foundation for the Promotion of Health and Biomedical Research in the Valencian Region (FISABIO), Valencia, Spain; 3 Department of Medicine and Surgery, Universidad Cardenal Herrera-CEU, CEU Universities, Valencia, Spain; 4 Austrian Chamber of Pharmacists, Upper Austria (APO), Linz, Austria; 5 Upper Austrian Society for General and Family Medicine (OBGAM), Linz, Austria; 6 Spanish Society of Family and Community Pharmacies (SEFAC), Madrid, Spain; 7 Spanish Society of Primary Care Physicians (SEMERGEN), Madrid, Spain; 8 Milladoiro Health Center, Santiago de Compostela Health Area, Madrid, Spain; 9 Cardiology Department, Klinikum Wels-Grieskirchen, Wels, Austria; 10 Instituto Pluridisciplinar y Facultad de Farmacia UCM, Madrid, Spain, Madrid, Spain; 11 IdISSC, Madrid, Spain; 12 Unidad de investigación de Atención Primaria de Salamanca (APISAL), Instituto de Investigación Biomédica de Salamanca (IBSAL), Salamanca, Spain; 13 La Source, School of Nursing Sciences, HES-SO University of Applied Sciences and Arts Western Switzerland, Lausanne, Switzerland; 14 AIT Austrian Institute of Technology, Center for Health & Bioresources, Medical Signal Analysis, Vienna, Austria; 15 H&TRC - Health & Technology Research Center, Coimbra Health School, Polytechnic University of Coimbra, Coimbra, Portugal; 16 Department of Applied Systems Research and Statistics, Johannes Kepler University, Linz, Austria

**Keywords:** arterial stiffness, estimated pulse wave velocity, community pharmacies, primary care, hypertension, vascular age, team-based approach

## Abstract

**Background:**

Highlighting arterial stiffness in Community Pharmacies (CPh) has been met with considerable interest in Portugal, Austria, and Spain. TOGETHER aims to evaluate whether empowering hypertensive subjects by determining blood pressure (BP) and vascular aging in CPh increases hypertension (HTN) control, while establishing paths of lasting cooperation between General Practitioners (GP) and CPh.

**Methods:**

TOGETHER is a cluster-randomized, prospective study in Portugal, Austria, and Spain. All consecutive subjects entering CPh will be offered BP measurement and ambulatory BP monitoring (ABPM) for those with BP ≥ 140/90 mmHg. CPh will be randomly assigned to a usual care arm (including health education for HTN). In the experimental care group, vascular aging (VA) will be additionally assessed by estimating aortic pulse wave velocity using brachial oscillometry. In this group, health education will include VA, which will also be communicated to GPs. In both groups, HTN will be treated by GPs according to usual clinical practice. A second evaluation with ABPM will be performed after 6 months, and the percentage of patients with controlled hypertension will be compared between both arms. The degree of CPh/GP interaction and patients´ adherence will be assessed through validated surveys.

**Discussion:**

Five fundamental unmet needs will be addressed by TOGETHER: 1) the lack of HTN screening programs; 2) implementation of the simpler and more informative VA concept (“Your arteries are 10 years older than you”); 3) understanding CV risk on an individual basis (individual VA) should improve patient adherence and reduce physician inertia; 4) implementation of health educational programs, combined with the concept of VA; 5) higher collaboration and development of a team-based approach to HTN care between CPh and GP. The complementary organizational settings and strengths of CPh and GP will allow TOGETHER to create new strategies for the early identification and intervention in uncontrolled HTN, encompassing both lifestyle and medical treatment, while considering social, economic, and behavioral aspects.

**Clinical trial registration:**

https://clinicaltrials.gov/, identifier FP-1533-ROD-SAL-2024-01.

## Introduction: the five challenges in controlling high blood pressure

### Poor control of hypertension and absence of sustained screening strategies

Cardiovascular (CV) diseases are the leading cause of death in Europe, with 336.4 deaths/100,000 inhabitants, clearly ahead of cancer with 235.3 deaths/100,000 inhabitants/year in 2021. Among the three different countries participating in TOGETHER, Austria (AT), Portugal (PT), and Spain (ES), AT ranks first with 343.4 CV deaths/100,000 inhabitants/year, followed by PT and ES with 247.9 and 213.0 CV deaths/100.000 inhabitants/year, respectively (1).

High blood pressure (BP), known as arterial hypertension (HTN), is the leading risk factor for CV diseases (2). Unfortunately, reliable data on the prevalence and treatment of HTN in the three participating countries are scarce. In May 2017, as part of the International Society of Hypertension (ISH) “May Measurement Month”-campaign, standardized BP measurements were conducted in 80 countries around the world. In AT (3), a total of 2,711 people were screened for HTN. Of these, 1,704 (62.9%) had BP values > 140/90 mmHg. The result for people who were already taking antihypertensive therapy was particularly worrying as 63.5% had uncontrolled HTN, while 43.2% of subjects without antihypertensive treatment were hypertensive.

The results of the same MMM-2017 campaign in ES were similar. A total of 7,646 subjects participated throughout ES; 40.0% presented with HTN, of which 74.4% were aware of their disease, while up to 25.6% of patients with high BP levels were unaware of their HTN status. Among participants who were not taking antihypertensive drugs, elevated values were measured in 16.9%. In treated hypertensive patients, 36.4% had uncontrolled values (4).

In PT, the prevalence of HTN in the adult population was estimated to be 42.2% in the PHYSA study (5), a representative sample of the 18–90-year-old population, including 3,720 participants, with 70% of the HTN patients under pharmacological medication. The age-specific prevalence of hypertension was 6.8, 46.9, and 74.9% in people below 35 years, 35–64 years, and above 64 years, and overall, among the hypertensive patients, 76.6% were aware of the HTN condition, 74.9% were treated, but only 42.5% were controlled.

Despite the enormous significance of HTN and the very low rates of control of elevated BP, there are currently no established population-screening programs aimed at diagnosing HTN in most countries around the world, including PT, AT, and ES.

### Deficiencies in estimating cardiovascular risk using risk tables

The implementation of CV risk stratification has represented a significant milestone in the prevention of CV diseases (6–8). The concept of subclinical target organ damage (TOD) was introduced in 1999 as a parameter that modifies the prognosis of patients independently and additively to the presence of CV risk factors to improve risk stratification (9–11). The latest ESH Guidelines recommend the determination of TOD whenever possible to aid in the management of hypertensive patients (12). Hypertension-mediated organ damage (HMOD) may involve different organs, such as left ventricular hypertrophy at the cardiac level, increased urinary albumin excretion, and reduced glomerular filtration rate at the renal level. However, these assessments are difficult to determine in large-scale HTN screening campaigns.

The most widespread and known HMOD in the vascular bed is atherosclerosis, an alteration that mainly affects the arterial lumen. A different HMOD is called arteriosclerosis, a condition in which mainly the arterial wall and pulse wave propagation characteristics are altered. Both terms, “atherosclerosis” and “arteriosclerosis,” are frequently used in the literature as synonyms. Although they often overlap and share risk factors, these are different pathophysiological entities. Arteriosclerosis is mainly due to the replacement of elastin fibers by collagen of a more rigid nature and complex changes of muscle fibers in the media. These changes increase pulse wave velocity (PWV), evidencing increased arterial stiffness, and contribute to isolated systolic HTN, elevated pulse pressure, and accelerated (early) vascular aging (EVA). Atherosclerosis can be diagnosed by ultrasound of superficial arteries, but this technique is limited to specialized centers and is unsuitable for large-scale population screening.

The gold standard method for assessing arteriosclerosis (arterial stiffness) is carotid-femoral applanation tonometry (13). It is a complex technique that requires training, time, and equipment not currently affordable to perform a population-based screening. The AIT Austrian Institute of Technology (AIT, Vienna) and collaborators have developed and validated a new method for estimating aortic PWV from the pressure wave morphology, blood pressure, and age in recent years (14).

Validation studies (15–17), the establishment of normal values in different European countries (18, 19) (Supplementary Figure 1) and extensive experience in various countries (20–22) makes PWV estimation from brachial arterial BP cuffs a viable option for detecting subclinical vascular damage in large-scale clinical studies where conventional measurement of PWV is not feasible (e.g., screening in pharmacies or public spaces), but does not substitute the actual measurement of cfPWV.

Vascular age (VA) can be defined by age-adjusted percentiles of estimated PWV (ePWV) assessed with oscillometry, because ePWV shows a monotonous increase with age. In other words, higher VA means the ePWV is above the 90th percentile for the corresponding age group, and lower VA means the ePWV is below the 10th percentile for the corresponding age group (Supplementary Figure 1) (18). The calculation of the vascular age (VA) based on ePWV is done by a proprietary algorithm by IEM provided by the HMS Client as software for Tel-O-Graph and Mobil-O-Graph. According to the manufacturer’s manual,^1^ it is determined from the hemodynamic information and based on the normal age-related development of arterial stiffness in healthy people, measured in meters per second. Following this reasoning in arterial damage, the concept of “early vascular aging (EVA)” has been developed in recent years, i.e., accelerated vascular aging, suggesting that there is a difference between chronological age, defined by date of birth, and biological age, characterized by the actual state of the vascular tree. There is no consensus about the classification of EVA in the literature, but the 90th percentile of age-adjusted PWV is reasonable and commonly used. Arterial wall alterations measured in practice can therefore be translated into concepts such as arterial aging as a tool for early screening and motivating individuals to seek medical evaluation.

In summary, by estimating arterial stiffness through brachial oscillometry, it may be possible to diagnose subclinical arterial damage, translated into the concept of EVA.

### Difficulties in understanding the traditional concept of “cardiovascular risk”

CV risk stratification represents a theoretical concept that generally lies outside the scope of understanding for most citizens. EVA, estimated as PWV (ePWV) by brachial oscillometry, offers a simpler alternative (19). As a result, it is now possible to calculate for each subject individually, if the measured VA value is below the 50th percentile, between the 50th and 90th percentile, or above the 90th percentile of normal values. A simple graphic representation in the form of the system of traffic lights (red–yellow–green) serves for the patient to understand the measured value and obtain a direct visual reference of the state of his arterial system (Supplementary Figure 2), similar to the risk associated with food intake based on its fat content in some European countries.

In the last years, different feasibility studies have been published independently in three European countries, showing that the concept of VA/EVA could be successfully implemented in community pharmacies (CPh) (20–22).

### Deficiencies in structured health education of hypertensive subjects

Health education represents an effective measure to control HTN, regardless of whether it is carried out in health centers, medical consultations, or CPh (23). Unfortunately, there are no systematic educational programs related to HTN in PT, AT, and ES yet. One recent study (24) confirmed that motivating patients to adopt healthier lifestyles by communicating their Heart-Age, according to the so-called Heart-Age tool, was superior to the traditional percentage-based risk tables. Bakhit et al. (25) published recently a meta-analysis including 62 randomized trials that compared any CV disease risk communication strategy versus usual care in terms of accuracy of risk perception, clinician-reported changes in CV risk, psychological responses, intention to modify lifestyle, self-reported changes in risk factors and clinician prescribing of preventive medicine, showing that any kind of risk communication led to a small but significant BP reduction, mainly by improvement of the CV risk perception and increase in medication adherence.

Many studies have demonstrated the relevant role of the CPh in both the primary and secondary CV prevention (26–28). Many of these interventions included monthly follow-up focused on adherence to nutritional guidelines and modification of lifestyle habits (physical exercise and smoking cessation), as well as patient education, counseling about lifestyle, feedback to physicians, medication adherence, and medication management. Evidence from systematic reviews with meta-analyses supports that pharmacist interventions, alone or in collaboration with other healthcare professionals, improve BP management (29–32). Recent guidelines on HTN, notably the 2017 guidelines from the American College of Cardiology and the American Heart Association, as well as the 2023 guidelines of the ESH, recommend the involvement of pharmacists for the team-based management of HTN (12, 33).

### Absence of structured communication between general practitioners (GP) and community pharmacies (CPh)

General practitioners (GP) and CPh represent two key actors in the management of all healthcare systems. Several initiatives document the willingness of collaboration between both groups, specifically in the field of HTN (34–36); however, there are no public or private programs integrating these two actors of health systems in an institutional way, especially not for the control of specific pathologies, such as HTN. The literature has described validated surveys that analyze the relationship between pharmacists and GP, but their implementation is limited to the scope of research and does not yet form part of daily clinical and pharmaceutical practice (37).

The objective of this study is to develop collaborative programs aimed at lightening the burden of primary care, improving health education shared between CPh and GP, whether in health centers or private consultations, and to improve both the early diagnosis of HTN and its control.

## Methods

### Participants

The study will include CPh customers, following written informed consent, who volunteer to participate and fulfill the inclusion criteria (Table 1).

**Table 1:** Inclusion and exclusion criteria.

Inclusion criteria
Age ≥ 18 years
BP ≥ 140 mmHg and/or ≥ 90 mmHg in the pharmacy
24-h ABPM ≥ 130 mmHg or ≥ 80 mmHg
Willingness to fill in forms, regarding
Anamnesis (DM, dyslipidemia)
Toxic habits
Pharmacological groups of CV medication
Previous CV diseases
Data on GP and assigned health center
Survey regarding interaction with GP and community pharmacist

Exclusion criteria
Pregnancy
Inability to understand the project (language barrier)
Manifest incapacity or knowledge of the existence of a legal representative
Factors preventing the correct measurement of BP/VA (arrhythmias, arm circumference, etc.)
Previous measurement of VA in the last year

CPh will be selected based on previous experience with VA assessment and their contributions to research in the frame of scientific pharmacy societies. Each pharmacy is assigned a status of “rural” or “urban” at the time of cluster randomization with 50,000 inhabitants as a threshold for the definition.

### Objectives

The main objective of this study is twofold: to evaluate whether a program including measurement and explanation of VA improves the interaction between CPh and GP, and whether it improves BP control as assessed by ABPM (Table 2).

**Table 2:** Main and secondary objectives: VA-based program leads to.

Main objectives
A more consistent interaction between community pharmacists and GP as indicated by the achievement of scheduled visits (endpoint: Visit to the Doctor, that is, how many hypertensive patients attend their recommended appointment with their family doctor?)
A better achievement of 24-h BP control, assessed with 24-h ambulatory BP monitoring in the “EVA cohort” compared to the “BP cohort” (endpoint: 24-h SBP/DBP < 130/80 mmHg, that is, how many hypertensive patients normalize their ABPM results?)

Secondary objectives
Reduction of blood pressure
Improvement of cardiovascular risk, vascular age, and risk factors
Better engagement primary care—community pharmacy—patients
Improvement of adherence to treatment and therapeutic inertia
Lack of differences between health systems
Optimization of the oscillometric pulse wave estimation algorithm

Secondary objectives include (i) assessment of improvement of office and 24-h BP, (ii) improvement of CV risk factors, VA and CV risk, (iii) improvement of the relationship between pharmacies, GP and patients, (iv) assessment of changes on the side of the patients, and (v) improved therapeutic inertia on the side of the GP (Table 2) through measurement and explanation of VA as compared to “usual care.” In this study, “usual care” refers to an already optimized situation—as compared to standard care—that attempts to provide the best possible follow-up that hypertensive patients receive in their healthcare system using current methods (i.e., excluding VA), including a dedicated educational and empowerment program for patients. Finally, data obtained in TOGETHER will also be used for the optimization of the oscillometric pulse wave estimation algorithm as a secondary objective.

Because the TOGETHER project is carried out simultaneously in two healthcare systems with different organizational structures, it will be appropriate to distinguish the care in ES/PT (universal coverage, financed by the government through tax payments, care predominantly provided within the public sector and free of charge at Primary Care Centers) from that given in AT (universal compulsory coverage, provided either through statutory health insurance or substitutive private health insurance, care provided mainly within private offices also free of charge). Both scenarios share the design of a “BP cohort” with usual care as defined *a priori*. We hypothesize that differences in the healthcare structure of AT, PT, and ES will not change the effect of a VA-based program for HTN management HTN when compared to the usual care (Table 2).

### Design

**Figure 1:**
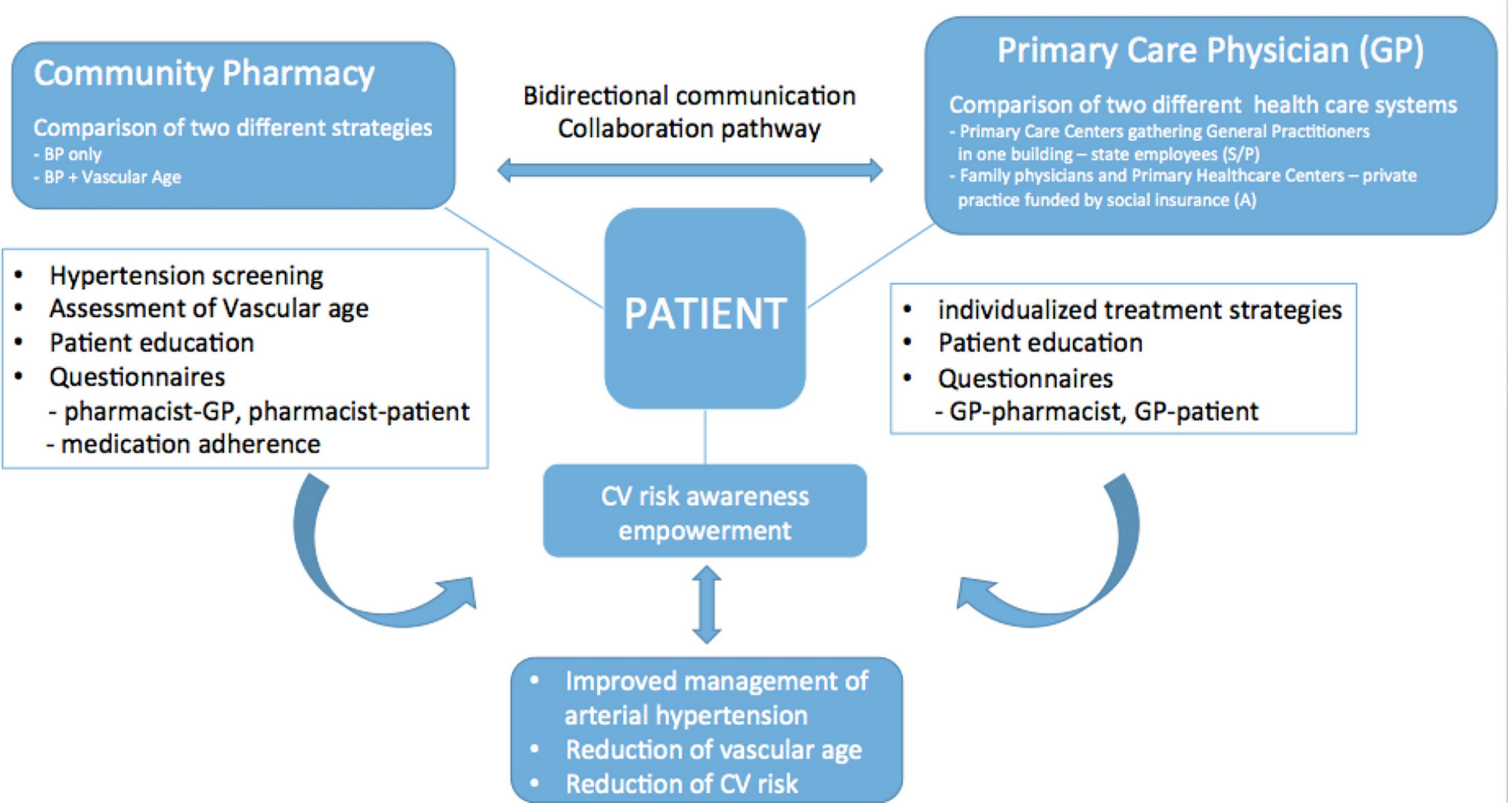
Representation of the interaction between community pharmacies and general practitioners.

This protocol is based on the hypothesis that the concept of “Vascular Age,” being more accessible and easy to grasp than other medical terms, will help hypertensive patients to better understand their CV risk. Our hypothesis assumes that this awareness of the state of their health will empower them to take actions in pursuit of its improvement, resulting in a better interaction with healthcare providers as well as in a better control of their BP compared to patients who received the usual medical care (Figure 1).

Likewise, we think that the empowerment of patients through knowledge of their VA will establish a link of greater trust between GP and the CPh participating in the study, compared with usual care. The TOGETHER Project has a longitudinal, cluster-randomized, prospective design with two cohorts:

BP cohort: This is the usual care cohort. At the baseline visit, BP will be measured, followed by usual care. ePWV will also be determined in a blinded fashion (for comparison of the baseline variables), but the value will not be communicated to the pharmacist, the GP, or to the patient until the end of the study.

EVA cohort: As in the BP cohort, BP will be measured at baseline. In addition, ePWV from brachial oscillometry will be assessed to determine VA, which will be communicated to the patient, the pharmacist, and the GP.

After stratification by region, each participating CPh will be assigned a cohort type randomly, providing a homogeneous geographical distribution so that ~50% of pharmacies will communicate only BP (BP cohort) and 50% will communicate BP and vascular age (EVA cohort), using the strategy of cluster randomization. Cluster randomization is performed region-specifically according to established standard operating procedures (SOPs). Participating centers are stratified (by urban/rural location, local competition, and size) and then randomized to form two blocks. These blocks are designed to contain as equal a number of health centers as possible and are then randomly assigned to EVA or BP. To permanently prevent selection bias, the SOPs also include specific guidelines for any future acquisition of centers, ensuring that cohort assignment is unpredictable. No inclusion number per pharmacy is set *a priori*. The number of patient inclusions will be monitored specifically for each country. After the specified total number of participants in a country has been reached, the pharmacies are informed that the acquisition of study participants must be stopped. The expected recruitment time will be at least 6 months.

### Primary and secondary outcomes

The following primary and secondary endpoints are defined (Table 3):

1) Visit to the doctor [yes/no]:
  Proportion of patients in the EVA cohort who comply with the visit to the Primary Care Physician (GP) compared to the proportion of patients in the BP cohort who comply with the visit to the Primary Care Physician.
2) Normalization of ABPM after 6 months (defined as average 24-h BP < 130/<80 mmHg) [yes/no]:
  Proportion of hypertensive patients in the EVA cohort who normalize 24-h blood pressure compared to the proportion of hypertensive patients in the BP cohort who normalize 24-h blood pressure as assessed by a second ABPM 6 months after the first ABPM.

**Table 3:** Primary and secondary outcomes.

Primary outcomes
Visit to the doctor [yes/no] Proportion of patients in the EVA cohort who comply with the visit to the Primary Care Physician compared to the proportion of patients in the BP cohort who comply with the visit to the Primary Care Physician.
Normalization of ABPM after 6 months <130/<80 mmHg [yes/no] Proportion of hypertensive patients in the EVA cohort who normalize blood pressure compared to the proportion of hypertensive patients in the BP cohort who normalize blood pressure as assessed by a second ABPM 6months after the first ABPM.

Secondary outcomes
Achievement of the last scheduled visit for carrying out the second ABPM (ES/PT): in the pharmacies, AT: doctor’s practice) [yes/no]
Reduction of 24-h SBP by 5 mmHg and/or DBP by 2 mmHg [yes/no]
Absolute changes in 24-h SBP mmHg
Absolute changes in 24-h DBP mm Hg
Changes in ePWV [m/s] and VA [years compared to expected age]
Changes in lipid metabolism (total cholesterol, LDL cholesterol [mg/dl])
Changes in weight [kg] and waist circumference [cm]
Changes in smoking habits (smoking status [yes/no])
Changes in vascular risk stratification by SCORE (ES/PT) and SCORE2 (AT) [%]
Changes in the score of collaborative surveys between pharmacists, doctors, and patients
Compliance with prescribed antihypertensive treatment [optimal if ≥ 80%/mean if ≥ 50% and < 80%/insufficient if < 50%]

### Procedures

**Figure 2:**
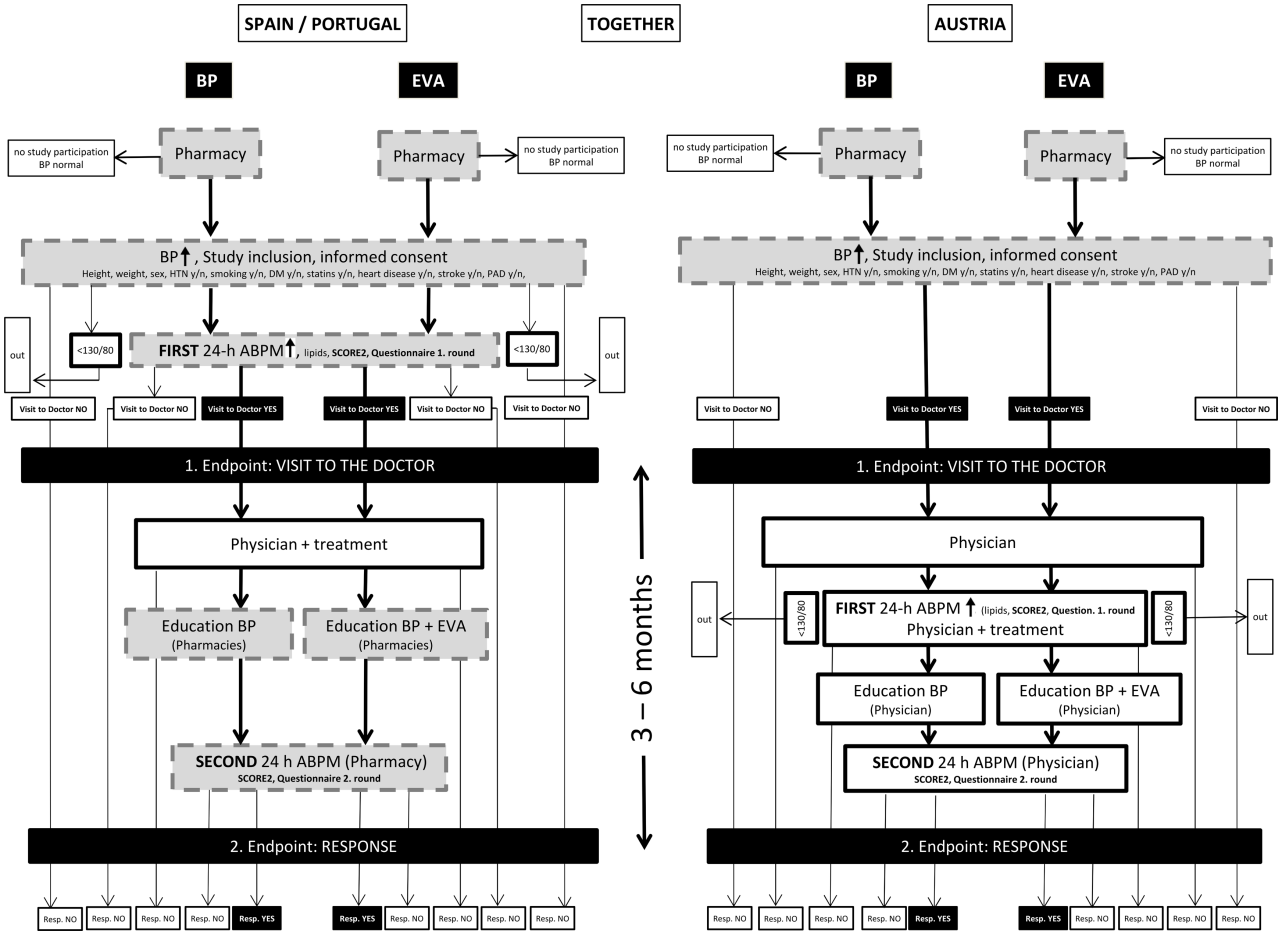
Study flowchart (for a more detailed explanation, see Supplementary Figures 3, 4A–D).

All study procedures are in accordance with recent HTN guidelines (12). The Study Flowchart describes how the two primary endpoints can be achieved (Figure 2). Figure 2 is divided into two parts: ES/PT on the left and AT on the right. In turn, each part includes the two cohorts: “BP” (usual care) and “EVA” (usual care + vascular age). Gray boxes with dotted edges represent CPh, white boxes with smooth black edges represent GP, indicating where the study procedures described inside take place. The two main endpoints are located at the same level in a box with a black background. Likewise, patients who have been complying with the planned visits are identified in black boxes, while those who have not done so and/or do not reach the control objective (ABPM in the second visit < 130/80 mmHg) appear in boxes with a white background. Note how in ES/PT the visit to the doctor takes place according to protocol only once, while in AT it is the pharmacy that intervenes once in the first visit, expressing the structural differences between both health systems.

### Procedures in detail

The specific steps of this process are summarized in Table 4. Recruitment of CPh takes place through previous collaborations and/or invitation from public institutions, either the Austrian Chamber of Pharmacists/Upper Austria (APO), or scientific societies such as SEFAC (Spanish Society of Family and CPh) in Spain. The random allocation of the CPh to the BP cohort or the EVA cohort follows cluster randomization, which takes place according to criteria of regional homogeneity.

**Table 4:** Procedures.

Spain/Portugal (ES/PT)
Community pharmacies
*Visit 1*: BP measurement, VA estimation, height, weight, questionnaires, informed consent, recruitment
*Visit 2*: ABPM, lipids, SCORE2, glucose metabolism, renal function
*Visit 4*: Lifestyle counseling
*Visit 5*: BP measurement, VA estimation, height, weight, questionnaires, ABPM lipids, SCORE2, glucose metabolism, renal function
General practitioner (GP)
*Visit 3*: Treatment, prescription if needed

Austria (AT)
Community pharmacies
*Visit 1*: BP measurement, VA estimation, height, weight, questionnaires, informed consent, recruitment
General practitioner (GP)
*Visit 2*: ABPM, lipids, SCORE2, glucose metabolism, renal function
*Visit 3*: Treatment, prescription if needed
*Visit 4*: Lifestyle counseling
*Visit 5*: ABPM, lipids, SCORE2, questionnaires

Trained pharmacy personnel will measure BP in CPh with a validated device, Tel-O-Graph® (IEM, Stollberg, Germany), according to a standard procedure following the 2023 European Society of Hypertension Guidelines for the Management of Arterial Hypertension (13), using automated attended triple measurement, and the BP shown is the average of the second and third measurement. In addition, the estimation of PWV and VA by brachial oscillometry with the same device, Tel-O-Graph® (IEM, Stollberg, Germany), will be performed in both the BP and the EVA cohort. In the BP cohort, this will be done in a blinded way, and the results will not be disclosed to patient, pharmacist, or physician. In the EVA cohort, the results will be shown to the pharmacist, discussed with the patients, and reported to the physician. The Mobil-O-Graph includes the same algorithm as the Tel-O-Graph to estimate ePWV, but in this study, it will only be used for 24-h brachial BP measurement.

Participants presenting with BP ≥ 140 mmHg and/or diastolic BP ≥ 90 mmHg will be invited to participate in the study. If the individual agrees and after explaining the aims of the study, study procedures, advantages, and disadvantages of participating, the informed consent (IC) will be signed and the individual will enter the study. Standardized education concerning HTN will then be given by trained pharmacy personnel in both cohorts (BP and EVA) in face-to-face meetings. In the EVA cohort, additional education about VA will be provided.

1) Austria
  Patients in both cohorts receive the recommendation to see their family doctor for further workup and, eventually, treatment of the elevated BP. Pharmacies prepare a report for the family doctor that contains the following:

- BP cohort: the result of the BP measured in the pharmacy.
- EVA cohort: the result of the BP measured in the pharmacy and VA.
  The GP performs ABPM, using the Mobil-O-Graph^®^ device (IEM, Stollberg, Germany) to confirm elevated BP. If ABPM BP is not elevated (24-h average SBP < 130 and DBP < 80 mm Hg), the patient will leave the study. If the BP is elevated, the GP will provide standardized BP education in both study cohorts. In addition, education is provided on the importance of VA and the prognostic consequences of increased VA in the EVA cohort. The education takes place in personal meetings or online by trained staff with the help of visual material that is adapted from ongoing BP training projects (herz.leben in Styria (38) PharmaFit in Madrid (26) and Team-Based Care (TBC-HTA) (39) in Switzerland. The standardized education on VA in the EVA cohort takes place in personal meetings in small groups by trained staff in the GP’s office using visual material adapted from the European COST (European Cooperation in Science and Technology) Action VascAgeNet (“Network for Research in Vascular Ageing”) (40–43). The Upper Austrian Society for General and Family Medicine (OBGAM) and the study advisor will carry out the training for specialized staff in GP offices.

2) Spain/Portugal
  After study inclusion and the first education about BP (both cohorts) or VA (EVA cohort only), ABPM is carried out in the pharmacies using the Mobil-O-Graph^®^ device (IEM, Stollberg, Germany). If ABPM BP is not elevated (24-h average SBP < 130 and DBP < 80 mm Hg), the patient will leave the study. If BP is elevated, standardized BP education will be provided in the health centers assigned to the pharmacies in both study cohorts. In addition, education is provided on the importance of VA and the prognostic consequences of increased VA in the EVA cohort. The education on BP and on VA will be standardized and identical to the training in AT. The scientific society SEMERGEN (Spanish Society of Primary Care Physicians) or the Primary Investigator will carry out the training for the specialist staff in the health centers. Pharmacies prepare a report for the family doctor that contains the following:
  - BP cohort: the result of the BP measured in the pharmacy.
  - EVA cohort: the result of the BP measured in the pharmacy and VA, the result of the laboratory measurement, and the result of SCORE/SCORE2.

A determination of the most important CV risk markers (total cholesterol in ES/PT and total cholesterol, LDL, HDL cholesterol, triglycerides in AT) is carried out either in the pharmacy (ES/PT) or in the doctor’s office (AT) as part of the clinical routine. This allows CV risk to be calculated according to the SCORE/SCORE2 calculations.

In both ES/PT and AT, patients are managed and treated by the GP in accordance with the ESH 2023 HTN Guidelines (12) and clinical experience. The GP can request further examinations that he/she considers appropriate and that are not part of the study, such as resting ECG and echocardiogram.

A second ABPM will be performed between 3 and 6 months after the first ABPM, in ES/PT in the CPh, and in AT by the GP. At the same time as the second ABPM, the following measurements are also carried out in ES/PT and transmitted to the family doctors. BP cohort: BP measured in the pharmacy, cholesterol levels, and calculation of SCORE/SCORE2; EVA cohort: BP measured in the pharmacy, cholesterol levels, and calculation of SCORE/SCORE2. At the same time as the second ABPM, office BP is measured in AT by the GP. The study ends with the second ABPM. The patients are further treated by their GP according to clinical routine. It is readily available to the family doctors in AT and will be sent to them in a report in ES/PT (see above).

At the beginning of the study, in all patients, pharmacists and GP will complete validated questionnaires to evaluate the interaction between them (37, 44), while patients will be given questionnaires to assess adherence (45). This will be repeated at the end of the study.

### Statistics and sample size calculation

The study has a confirmatory status and is based on a superiority approach with a comparison of two cohorts (EVA vs. BP) in three collectives (sub-collective AT; sub-collective ES/PT, total collective AT + ES/PT = AEP) and with two primary endpoints (response YES/NO; visit to the doctor YES/NO) of the same value. This means that six equivalent pairs of hypotheses will have to be tested:

- H0_1/2/3/4/5/6_: In AEP/AT/ ES/PT, the frequency of visits to the doctor/the response is not higher in EVA than in BP.
- H1_1/2/3/4/5/6_: In AEP/A/ES/PT, the frequency of visits to the doctor/the response is higher in EVA than in BP.

The sample size estimations (type I error = 0.415% one-sided, adjusted according to Bonferroni; type II error = 10%; Fisher’s exact test; G*Power Version 3.1.9.2) were based on the following expected frequencies (details see Supplementary Figures 4A–D):

- Visit to the doctor YES (%; EVA/BP): ES/PT: 83.706/60.411; AT: 70/50.
- Response YES (%; EVA/BP): ES/PT: 46.17/26.775; AT: 37.8/ 21.25.

Assuming a 30% dropout rate, the calculations resulted in requirements of approximately 560 inclusions in ES/PT (EVA and BP: *n* ~ 280 each) and approximately 690 in AT (EVA and BP: *n* ~ 345 each).

Hypothesis testing will be performed by logistic regression (dependent variables: cohort, sex, age, SBP, DBP, smoker, heart rate, diabetes, obesity, pulse pressure (SBP—DBP), prior stroke, prior myocardial infarction, kidney disease, and pharmacy location. Type I error preservation (2.5% one-sided) will be realized by a graph-based multiple test approach (46, 47).

## Discussion and perspectives

The present study analyses whether a team-based approach to hypertensive patients, consisting of collaboration between CPh and GP through communication of VA, can improve BP control. Furthermore, the relationship between pharmacists, family physicians, and patients is investigated. If successful, assessment of BP and VA in CPh would allow for a large-scale screening and better control of HTN, improvement of CV risk stratification, a better understanding of CV risk factors by empowered patients, and thereby improve adherence. It would also facilitate the implementation of systematic educational programs shared by GP and pharmacies, leading thereby to a structured collaboration between these two important stakeholders in the healthcare system. The TOGETHER trial is in line with recent meta-analyses demonstrating the capacity of pharmacies to successfully manage HTN (48, 49) and on the importance of empowering patients through a better understanding of their CV risk (24).

Strengths of this study are, first the participation of expert teams, as previously documented by three independent lines of research with very similar results, second, the *a priori* collaboration of scientific societies of GP and CPh in three different countries, and third, the comparison of two very differently structured health systems. The limitations of this study are the focus on a widespread and easy-to-use oscillometric technique to estimate ePWV and VA due to the screening purpose in a real world, community pharmacy setting, a bias in the samples, as recruitment is not population based, and how to implement the transmission of results between CPh and GP.

In conclusion, we have developed a team-based protocol that integrates BP, VA, and empowerment of hypertensive patients as a starting point for developing simple, collaborative, and cost-effective interventions for controlling HTN.

## Ethics and dissemination

### Ethics approval and consent to participat

This project has been approved by the Ethics Committee for Clinical Research at the Hospital de Sagunto (ref: ROD-SAL-2024-01) and by ethics committees in the participating centers. The study has been registered at ClinicalTrials.gov (FP-1533-ROD-SAL-2024-01). All participants will be informed of the study characteristics, and data will be treated with complete confidentiality, according to the requirements of Data Protection Laws in the different countries.

### Dissemination plan

The data will be available to members of the research group, who will be primarily responsible for dissemination. In addition, the variables used in each manuscript will be available to the entire scientific community through a standard scientific repository. The results of the study will be published in peer-reviewed open-access scientific journals and presented at national and international scientific conferences. Likewise, appropriate dissemination will be carried out through social networks and other media and directly to the people participating in the study.

## Data Availability

The original contributions presented in the study are included in the article/Supplementary material; further inquiries can be directed to the corresponding author.

## Footnotes

1
https://medaval.ie/docs/manuals/IEM-Mobil-O-Graph-Manual.pdf

## Group member of TOGETHER-Project Research Group

Monika Aichberger (MA), Florian Ardelt (FA), Vicente Baixauli (VB), Sergio Cinza (SC), Kathrin Danninger (KD), María Soledad Fernández (MSF), Viktoria Gastens (VG), Luis García-Ortiz (LGO), Ana Karen Guzmán-Aguayo (AGA), Bernhard Hametner (BH), Antonela Jiménez (AJ), Valeria Kopytina (VK), Cristina Lugones-Sánchez (CLS), Christopher C. Mayer (CCM), Zuri Montalar (ZM), Telmo Pereira (TP), Enrique Rodilla (ER), Valérie Santschi (VS), Wolfgang Schimetta (WS), Thomas Weber (TW).
