# Supplementary material for: Vascular age as a key for a team-based approach to manage blood pressure bridging community pharmacists and primary healthcare physicians—The TOGETHER trial
Source: Front Public Health. 2026 Jan 14;13:1723100. doi: 10.3389/fpubh.2025.1723100 (PMC12847303; doi:10.3389/fpubh.2025.1723100)

Supplementary Figure 1: Normal values for ePWV[18].

Normotensives (n= 1064)	
**Age (years)**	**PWV (m/s)<br>Median (10 – 90 Percentile)**
< 30	4.9 (4.4–5.3)
30 - 39	5.5 (4.9–6.0)
40 - 49	6.4 (5.8–6.9)
50 - 59	7.5 (6.9–8.2)
60 - 69	8.9 (8.2–9.7)
70 - 79	10.6 (9.8–11.5)
> 79	12.7 (11.6–13.3)

Supplementary Figure 2: Traffic light representation of vascular age (VA) for patients.

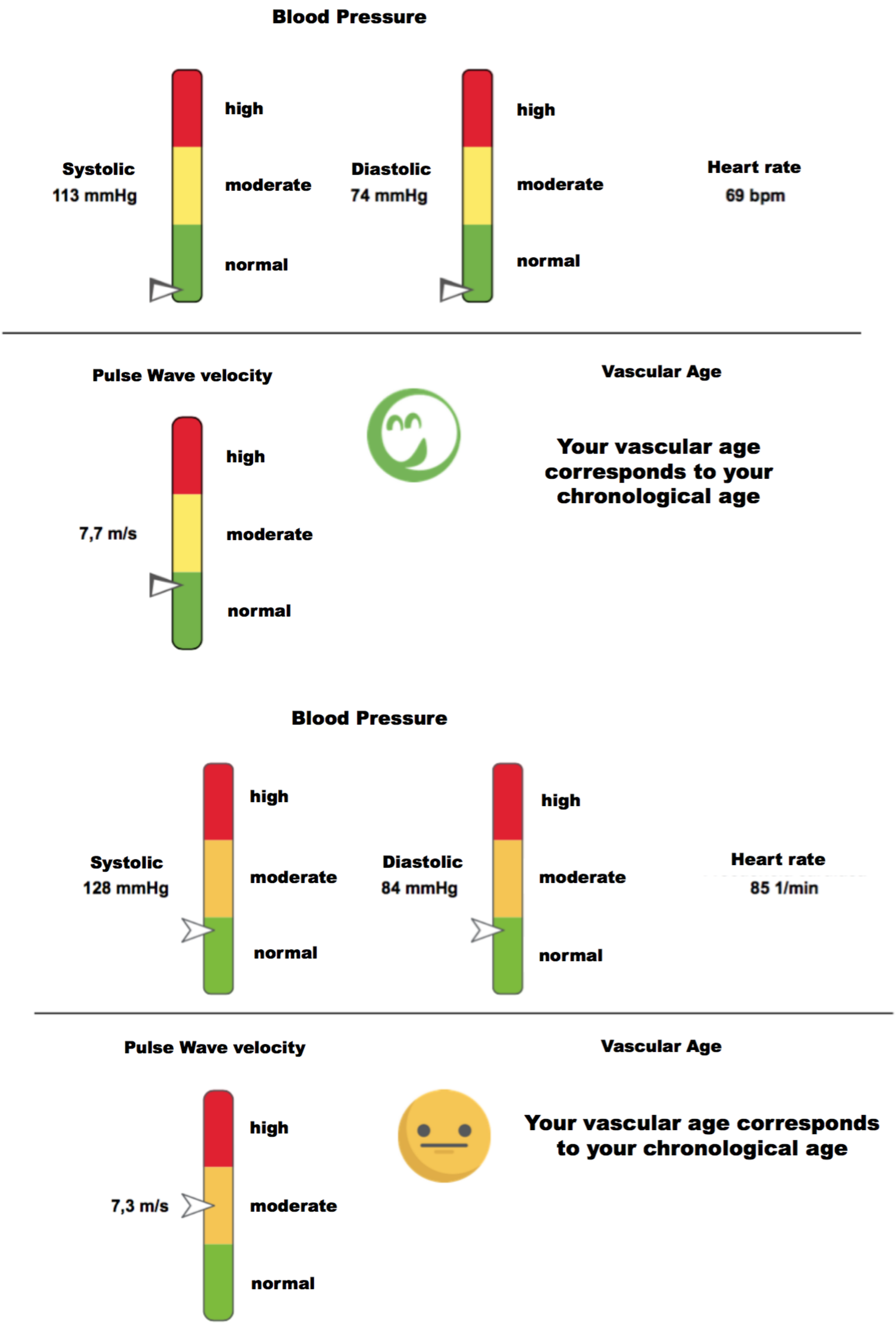

Blood Pressure

Systolic
150* mmHg

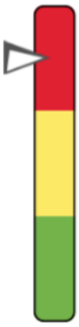

Diastolic
100* mmHg

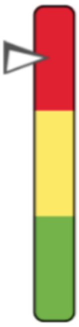

Heart rate
78 bpm

Pulse Wave velocity

9,8* m/s

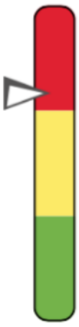

Vascular Age

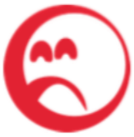

Your vascular age is older than your chronological age

Supplementary Figure 3: Flow Chart with detailed explanations.

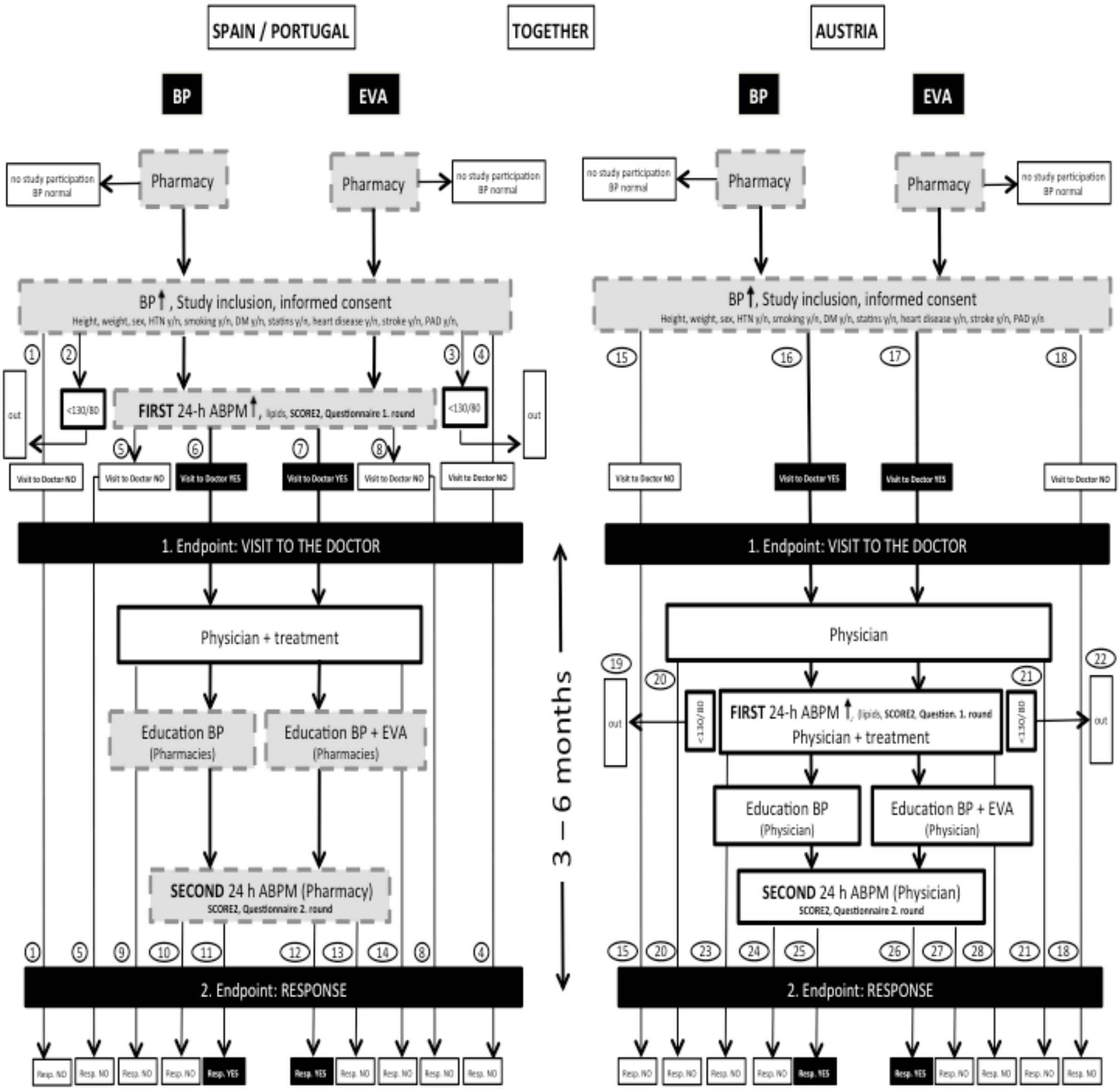

Description Study Flowchart

A) SPAIN / PORTUGAL

1 ... Pharmacy BP > 140/90 → study inclusion BP cohort → no ABPM → no visit to the doctor → BP response no

2 … Pharmacy BP > 140/90 → study inclusion BP cohort → ABPM < 130/80 → out

3 ... Pharmacy BP > 140/90 → study inclusion EVA cohort → ABPM < 130/80 → out

4 ... Pharmacy BP > 140/90 → study inclusion EVA cohort → no ABPM → no visit to the doctor → BP response no

5 ... Pharmacy BP > 140/90 → study inclusion BP cohort → ABPM > 130/80 → no visit to the doctor → BP response no

6 ... Pharmacy BP > 140/90 → study inclusion BP cohort → ABPM > 130/80 → visit to the doctor

7 ... Pharmacy BP > 140/90 → study inclusion EVA cohort → ABPM > 130/80 → visit to the doctor

8 ... Pharmacy BP > 140/90 → study inclusion EVA cohort → ABPM > 130/80 → no visit to the doctor

9 ... Pharmacy BP > 140/90 → study inclusion BP cohort → ABPM > 130/80 → visit to the doctor → lifestyle & treatment → no second ABPM (pharmacy) → BP response no

10 ... Pharmacy BP > 140/90 → study inclusion BP cohort → ABPM > 130/80 → visit to the doctor → lifestyle & treatment → second ABPM (pharmacy) → BP response no

11 ... Pharmacy BP > 140/90 → study inclusion BP cohort → ABPM > 130/80 → visit to the doctor → lifestyle & treatment → second ABPM (pharmacy) → BP response yes

12 ... Pharmacy BP > 140/90 → study inclusion EVA cohort → ABPM > 130/80 → visit to the doctor → lifestyle & treatment → second ABPM (pharmacy) → BP response yes

13 ... Pharmacy BP > 140/90 → study inclusion EVA cohort → ABPM > 130/80 → visit to the doctor → lifestyle & treatment → second ABPM (pharmacy) → BP response no

14 ... Pharmacy BP > 140/90 → study inclusion EVA cohort → ABPM > 130/80 → visit to the doctor → lifestyle & treatment → no second ABPM (pharmacy) → BP response no

B AUSTRIA

15 ... Pharmacy BP > 140/90 → study inclusion BP cohort → no visit to the doctor → BP response no

16 ... Pharmacy BP > 140/90 → study inclusion BP cohort → visit to the doctor

17 ... Pharmacy BP > 140/90 → study inclusion EVA cohort → visit to the doctor

18 ... Pharmacy BP > 140/90 → study inclusion EVA cohort → no visit to the doctor

19 … Pharmacy BP > 140/90 → study inclusion BP cohort → visit to the doctor → ABPM < 130/80 → out

20 … Pharmacy BP > 140/90 → study inclusion BP cohort → visit to the doctor → no ABPM → BP response no

21 … Pharmacy BP > 140/90 → study inclusion EVA cohort → visit to the doctor → no ABPM → BP response no

22 … Pharmacy BP > 140/90 → study inclusion EVA cohort → visit to the doctor → ABPM < 130/80 → out

23 … Pharmacy BP > 140/90 → study inclusion BP cohort → visit to the doctor → ABPM > 130/80 → lifestyle & treatment → no second ABPM → BP response no

24 … Pharmacy BP > 140/90 → study inclusion BP cohort → visit to the doctor → ABPM > 130/80 → lifestyle & treatment → second ABPM → BP response no

25 … Pharmacy BP > 140/90 → study inclusion BP cohort → visit to the doctor → ABPM > 130/80 → lifestyle & treatment → second ABPM → BP response yes

26 … Pharmacy BP > 140/90 → study inclusion EVA cohort → visit to the doctor → ABPM > 130/80 → lifestyle & treatment → second ABPM → BP response yes

27 … Pharmacy BP > 140/90 → study inclusion EVA cohort → visit to the doctor → ABPM > 130/80 → lifestyle & treatment → second ABPM → BP response no

28 … Pharmacy BP > 140/90 → study inclusion EVA cohort → visit to the doctor → ABPM > 130/80 → lifestyle & treatment → no second ABPM → BP response no

Supplementary Figure 4A: Estimated frequencies for primary endpoints.

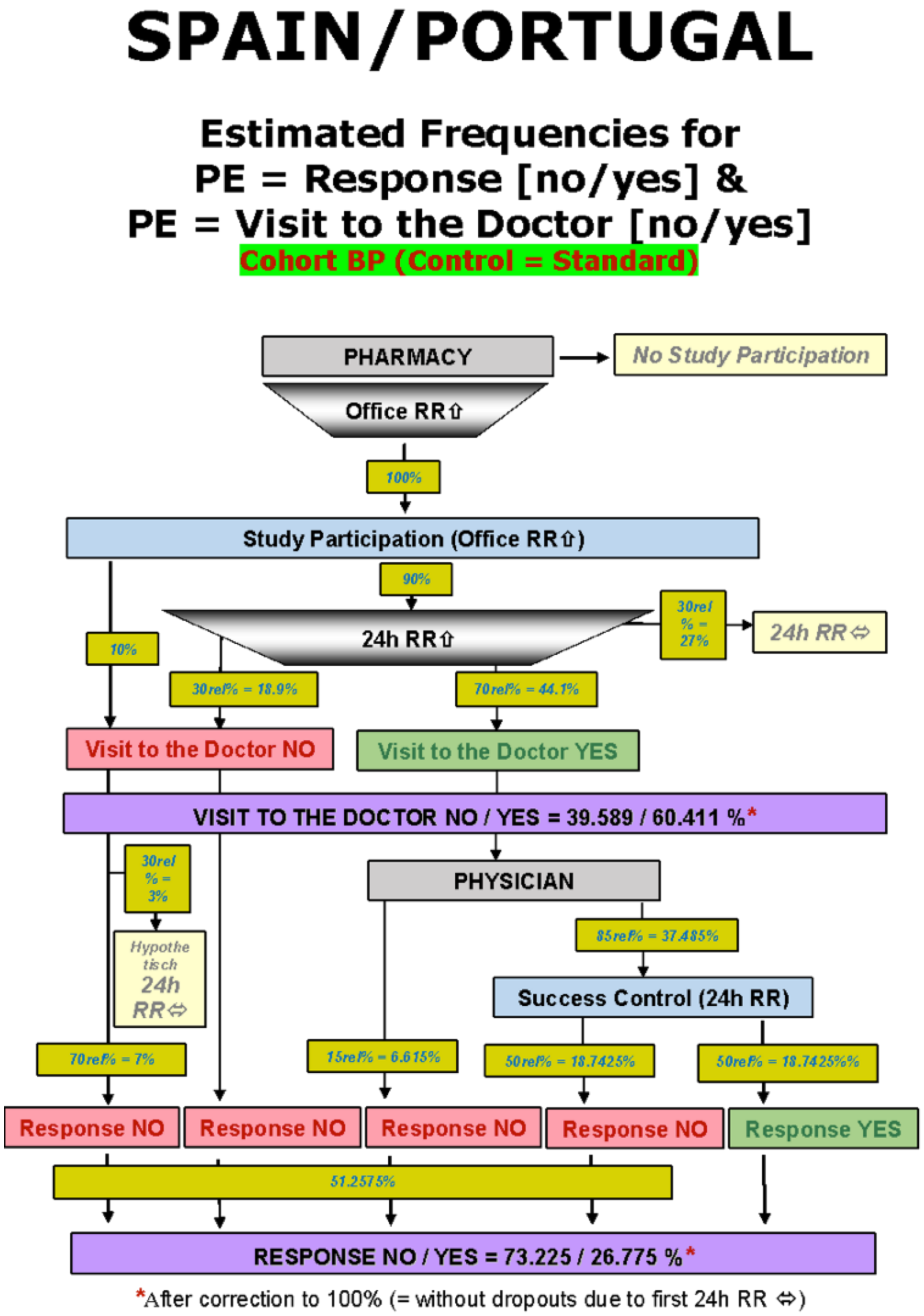

Supplementary Figure 4B: Estimated frequencies for primary endpoints.

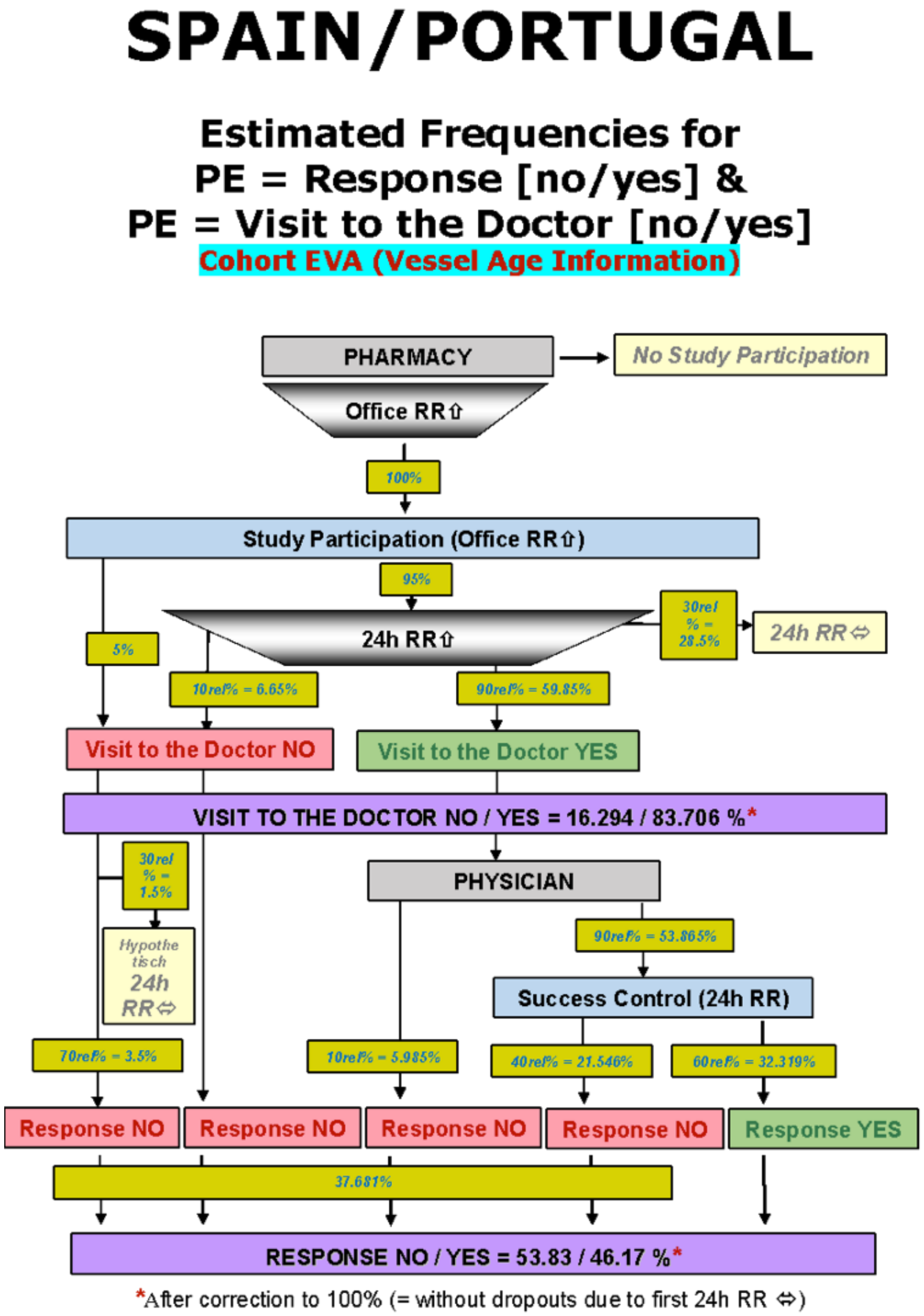

Supplementary Figure 4C: Estimated frequencies for primary endpoints.

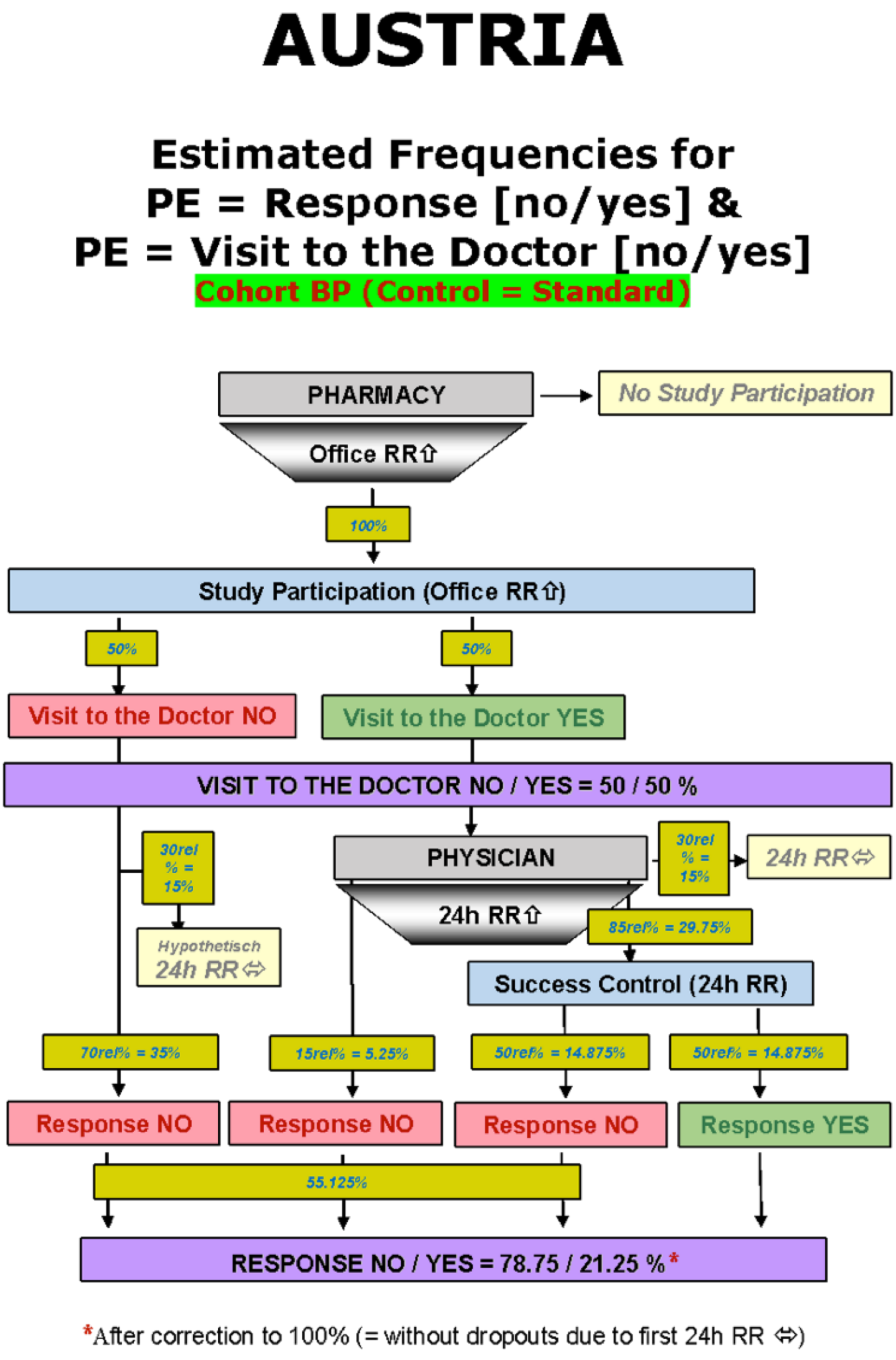

Supplementary Figure 4D: Estimated frequencies for primary endpoints.

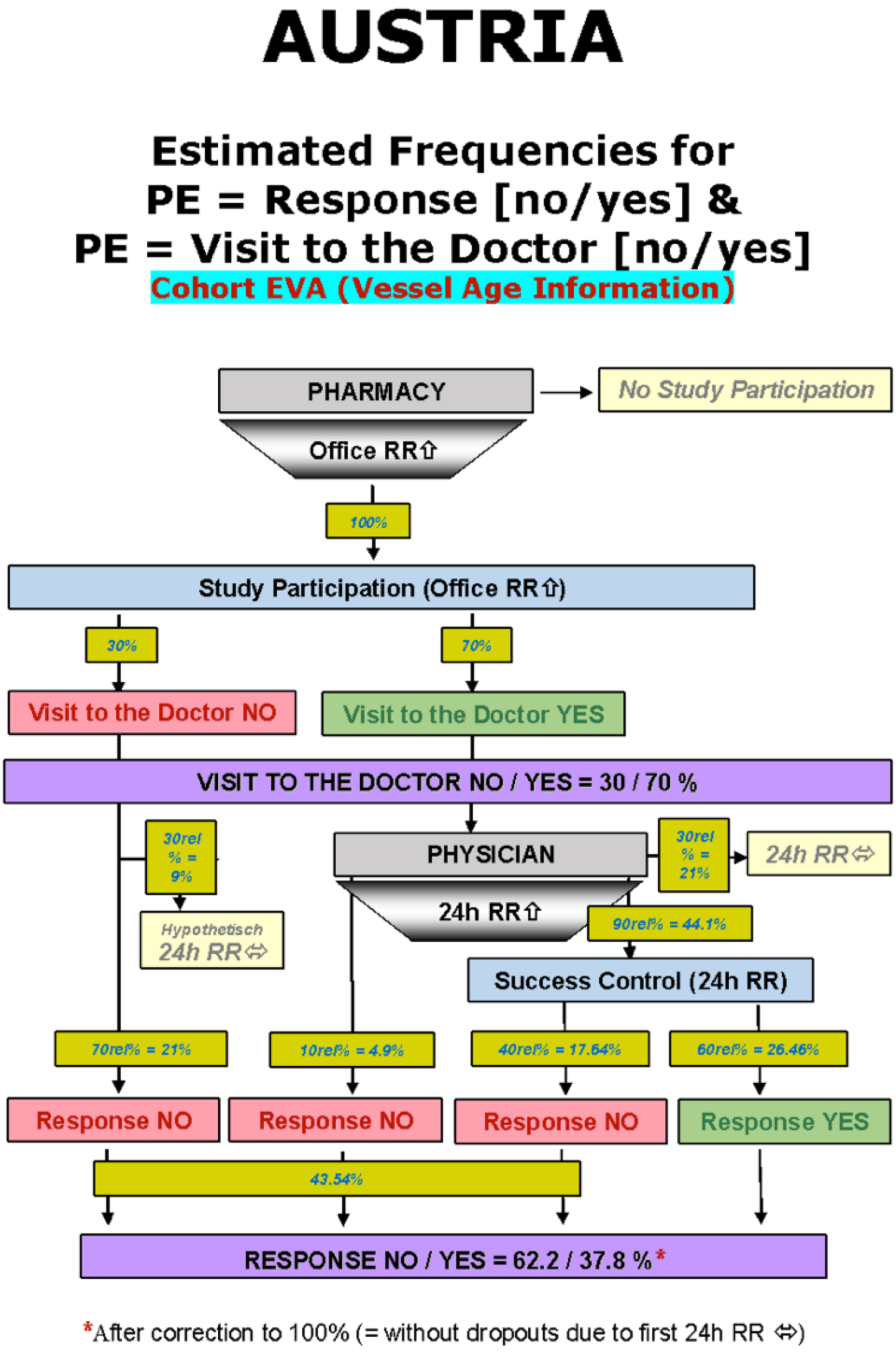

Supplement: Supplementary file 1 [file Data_Sheet_1.PDF]
